# Endothelial indoleamine 2,3-dioxygenase-1 regulates the placental vascular tone and is deficient in intrauterine growth restriction and pre-eclampsia

**DOI:** 10.1038/s41598-018-23896-0

**Published:** 2018-04-03

**Authors:** Pablo Zardoya-Laguardia, Astrid Blaschitz, Birgit Hirschmugl, Ingrid Lang, Sereina A. Herzog, Liudmila Nikitina, Martin Gauster, Martin Häusler, Mila Cervar-Zivkovic, Eva Karpf, Ghassan J. Maghzal, Chris P. Stanley, Roland Stocker, Christian Wadsack, Saša Frank, Peter Sedlmayr

**Affiliations:** 1 Gottfried Schatz Research Centre for Cell Signalling, Metabolism and Ageing, Department of Cell Biology, Histology and Embryology, Medical University of Graz, Graz, 8010 Austria; 2 Medical University of Graz, Department of Obstetrics and Gynaecology, Graz, 8036 Austria; 3 Medical University of Graz, Institute for Medical Informatics, Statistics and Documentation, Graz, 8036 Austria; 4 Medical University of Graz, Institute of Pathology, Graz, 8036 Austria; 5 Victor Chang Cardiac Research Institute, Darlinghurst, NSW 2010 Australia; 6 St Vincent’s Clinical School, UNSW Medicine, Sydney, NSW 2052 Australia; 7 Gottfried Schatz Research Centre for Cell Signalling, Metabolism and Ageing, Department of Molecular Biology and Biochemistry, Medical University of Graz, Graz, 8010 Austria

## Abstract

Indoleamine 2,3-dioxygenase-1 (IDO1) mediates the degradation of L-tryptophan (L-Trp) and is constitutively expressed in the chorionic vascular endothelium of the human placenta with highest levels in the microvasculature. Given that endothelial expression of IDO1 has been shown to regulate vascular tone and blood pressure in mice under the condition of systemic inflammation, we asked whether IDO1 is also involved in the regulation of placental blood flow and if yes, whether this function is potentially impaired in intrauterine growth restriction (IUGR) and pre-eclampsia (PE). In the large arteries of the chorionic plate L-Trp induced relaxation only after upregulation of IDO1 using interferon gamma and tumor necrosis factor alpha. However, *ex vivo* placental perfusion of pre-constricted cotyledonic vasculature with L-Trp decreases the vessel back pressure without prior IDO1 induction. Further to this finding, IDO1 protein expression and activity is reduced in IUGR and PE when compared to gestational age–matched control tissue. These data suggest that L-Trp catabolism plays a role in the regulation of placental vascular tone, a finding which is potentially linked to placental and fetal growth. In this context our data suggest that IDO1 deficiency is related to the pathogenesis of IUGR and PE.

## Introduction

L-Tryptophan (L-Trp) is the least abundant essential amino acid^1^. It is required for protein synthesis and also as precursor of key biomolecules such as serotonin, melatonin and NAD^+^ ^1–4^. L-Trp catabolism mainly follows the kynurenine (kyn) pathway (KP), with the first and rate-limiting step being the oxidation of the indole ring of L-Trp. This step is catalyzed by one of three enzymes capable of converting of L-Trp into N-formylkynurenine^5,6^, namely tryptophan 2,3-dioxygenase (TDO), indoleamine 2,3-dioxygenase-1 (IDO1) and −2 (IDO2). These cytosolic enzymes display varying expression patterns^2,7,8^, a factor which likely dictates their relative ability to contribute to the degradation of L-Trp along the KP.

It is well established that IDO1 is induced during infection where it displays antimicrobial activity^9^ and immune regulation^10,11^. Indeed, recent studies have probed the ability of the enzyme to mediate the immune system in tumour survival^12^. In the term placenta high levels of IDO1 expression and activity have been reported, where it is implicated in establishing and maintaining feto-maternal tolerance^13–16^, a role mediated via the induction of regulatory T cells^17^.

Other recent works have shown that during inflammation, IDO1 becomes expressed in vascular endothelial cells and contributes to arterial relaxation^18^. The enzyme is constitutively expressed on both the maternal and the chorionic vascular endothelium with a positive gradient towards the feto-maternal interface^13^. This led us to question whether expression of IDO1 in the fetal vessels of the chorion is involved in the regulation of placental perfusion and consequently placental and fetal growth. Furthermore, we wondered whether pathologies which display reduced fetal growth (such as intrauterine growth retardation (IUGR) and preeclampsia (PE))^19,20^, are associated with a defect in IDO1-mediated regulation of placental vascular tone. In fact, previous reports present some evidence in favour of this hypothesis. For instance, L-Trp degradation is reduced in placental tissue in IUGR^21^, and IDO1 expression is decreased in PE^22,23^. In addition, disruption of the IDO1 gene induces IUGR and preeclampsia phenotypes in pregnant mice^24^. Changes in the chorionic vasculature are associated with IUGR, as increased vascular resistance leads to abnormal Doppler waveforms of the umbilical artery^25^. Remodeling of the arteries of placental stem villi contributes to IUGR^26^. However, the role of IDO1 in the regulation of the placental vascular tone and its potential link with these pregnancy complications has not been investigated so far.

## Results

### Expression of IDO1 in isolated endothelial cells from chorionic plate arteries (PLAECs)

As a basis for subsequent functional studies involving chorionic plate arteries, we studied IDO1 expression at the level of mRNA and protein in freshly isolated primary PLAECs and in PLAECs stimulated with IFNγ (80 ng/mL) and TNFα (80 ng/mL) for 48 h^27^ (Supplementary Fig. S1). As revealed by qPCR, IDO1 mRNA expression was markedly augmented following cytokine stimulation (Supplementary Fig. S1a). IDO1 protein contents varied between the different isolations, and markedly increased upon cytokine stimulation (Supplementary Fig. S1b). The calculated MW of IDO1 is 45 kDa but the observed band was found at a somewhat lower level as previously described^9^. Immunocytochemical staining of cellular pellets failed to detect IDO1 protein in non-stimulated cells (Supplementary data, Fig. S1c1). However, strong IDO1-staining was found in PLAECs stimulated with IFNγ and TNFα (Supplementary Fig. S1c2).

### Effect of L-Trp on the relaxation of precontracted arterial rings of the chorionic plate

**Figure 1:**
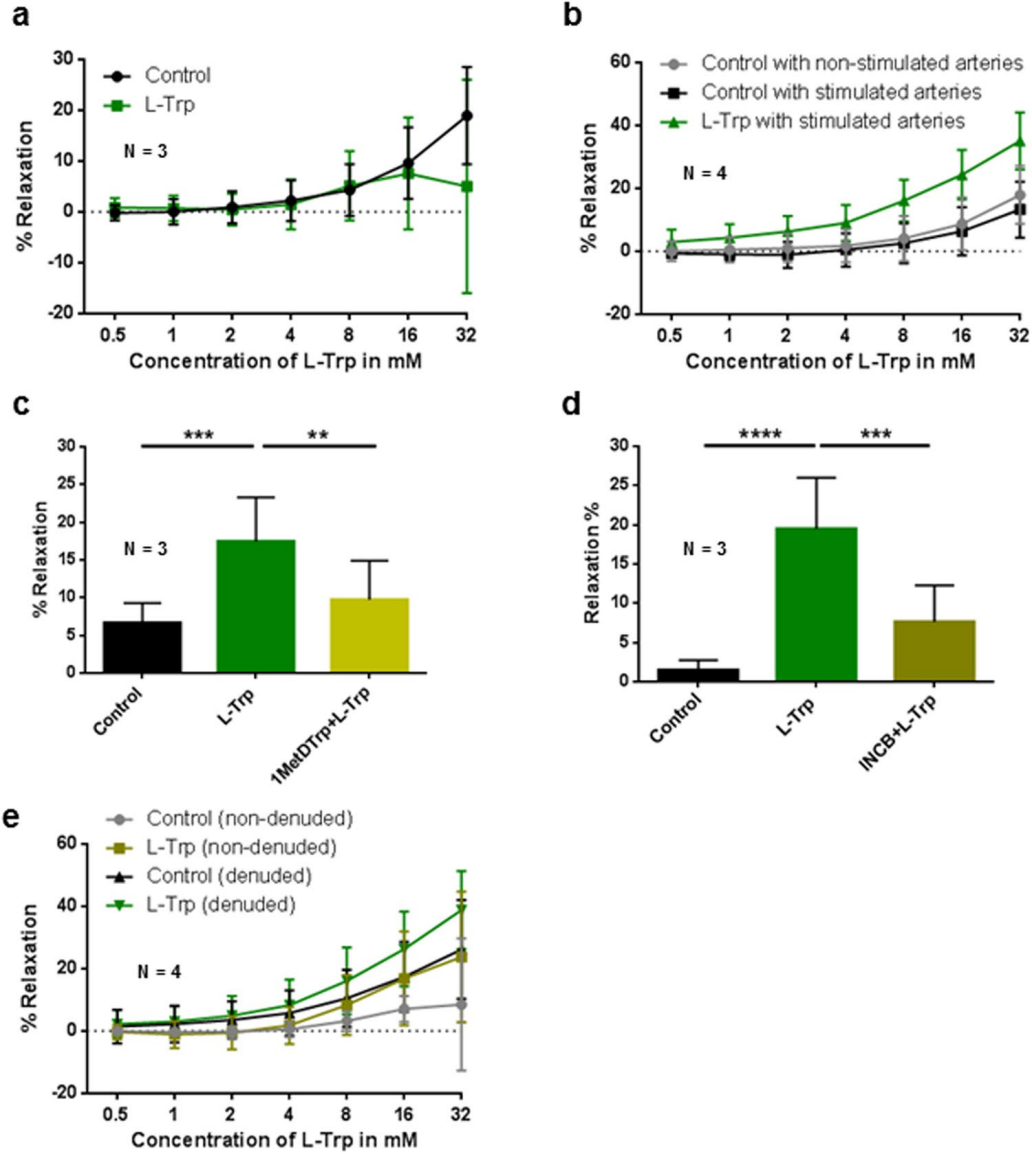
Impact of L-Trp on arterial rings of the chorionic plate of normal term placentas. The data show the magnitude of L-Trp- versus vehicle control- elicited relaxation of U46619-precontracted arterial rings pre-incubated overnight (**b**–**e**) or not (**a**) with TNFα and IFNγ. (**a**) Freshly isolated, non-stimulated arteries (P = 0.528). (**b**) Cytokine-stimulated arterial rings respond differently to L-Trp than stimulated and non-stimulated controls (P < 0.001); the controls did not react differently. (**c**) Relaxation following the application of 8 mM L-Trp in the presence (1MetDTrp + L-Trp; P ≤ 0.01) or absence (L-Trp; P ≤ 0.001) of 1 mM of the IDO-blocker 1MethylDTrp. (**d**) Relaxation following the application of 8 mM L-Trp in the presence (INCB + L-Trp; P ≤ 0.001) or absence (L-Trp; P ≤ 0.0001) of 30 μM of the IDO1-blocker INCB024360. (**e**) Reactivity of endothelium-denuded stimulated arterial rings to L-Trp differs from its (denuded) control group (P < 0.001). There is also a difference between the two control groups (denuded versus non-denuded, P = 0.034). Results are mean ± SD of at least 2 rings for each condition, obtained from at least 3 placentas; **P ≤ 0.01 (linear regression model in **a**,**b** and **e**; Dunnett’s test in **c** and **d**).

Compared to vehicle control, L-Trp had no discernible effects on U46619 pre-constricted arterial rings (P = 0.528) (Fig. 1a). In contrast, L-Trp- elicited relaxation of the arterial rings preincubated overnight with IFNγ and TNFα was significantly higher compared to vehicle-treated control rings (P < 0.001). Non-stimulated vehicle control arteries responded similar to stimulated control (P = 0.931) (Fig. 1b).

### The relaxing effect of L-Trp on stimulated term chorionic plate arteries is partly IDO1-dependent

To determine whether the relaxing effect of L-Trp depends on IDO1, we examined L-Trp-elicited relaxation of the rings preincubated with IFNγ and TNFα in the absence or presence of two different competitive inhibitors of IDO1, 1-methyl-D-tryptophan (Fig. 1c; 1MetDTrp) and INCB024360 (Fig. 1d; INCB024360). In comparison to vehicle treated control rings, L-Trp-elicited relaxation was significantly higher (Fig. 1c,d) (P ≤ 0.001 and P < 0.0001, resp.). Pre-treatment with either 1MetDTrp (P ≤ 0.01) (Fig. 1c) or INCB024369 (P ≤ 0.001) (Fig. 1d) resulted in reduced L-Trp-induced relaxation in stimulated rings.

### The effect of L-Trp on the vascular tone of stimulated chorionic plate arteries is not exclusively endothelium-dependent

To test the role of the endothelium in the L-Trp-elicited relaxation, myography was performed in rings with intact or denuded endothelium. The success of the denudation was assessed by immunohistochemistry (Supplementary Fig. S2). In denuded rings L-Trp elicited relaxation that was higher compared to (also denuded) control rings (P < 0.001) (Fig. 1e). We then performed myography measurements in the absence or presence of 1MetDTrp. Treatment with L-Trp relaxed the denuded arterial rings (P = 0.006, Supplementary Fig. S3). This effect was reduced by pre-incubation with 1MetDTrp (P = 0.0259), albeit to a small extent only (Supplementary Fig. S4). Therefore, our data indicate on one hand the presence of an IDO1-dependent pathway that catabolizes L-Trp when the endothelium is removed, on the other hand they suggest that there may be an additional element of IDO1-independent relaxation in denuded rings. Immunohistochemistry of a cytokine-stimulated artery ruled out an expression of IDO1 in smooth muscle cells, however, a few isolated cells in the vessel wall (possibly macrophages) stained positive for IDO1 (Supplementary Fig. S5).

### Impact of L-Trp on the vessel back pressure of a placental cotyledon

**Figure 2:**
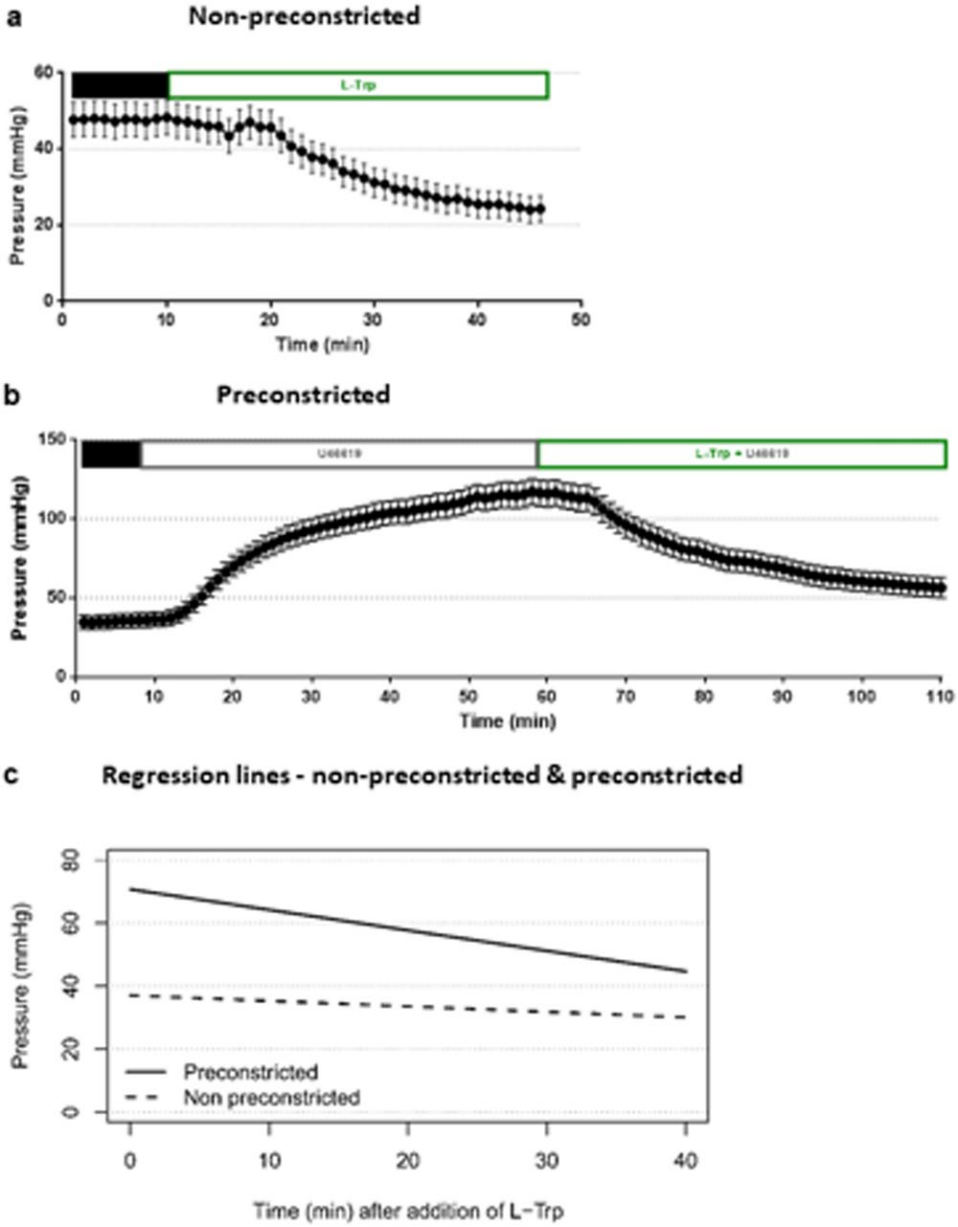
Impact of L-Trp on the vessel back pressure in placental cotyledons. (**a**) Representative experiment from a set of 5 independent experiments showing the time course of the vessel back pressure during perfusion with 8 mM L-Trp, after attaining a phase (10 min) of stable pressure (black bar). (**b**) Representative experiment from a set of 6 independent experiments showing the time course after attaining a phase of stable pressure (black bar) followed by perfusion of the cotyledon with U46619 (10 nM). After attaining stable (10 min) vessel back pressure again, the cotyledons were perfused with L-Trp (8 mM) and U46619 (10 nM). Each pressure point shows the average of 60 measurements. Results are given as mean ± SD. (**c**) Linear regression model. In both sets of experiments the decrease of the vessel back pressure during perfusion with 8 mM L-Trp is significant. Without preconstriction (dashed line, 5 experiments), the coefficient for the linear relationship between pressure and time is −0.175 (SE 0.020, P < 0.001). Following preconstriction (continuous line, 6 experiments), the coefficient for the linear relationship between pressure and time is −0.651 (SE 0.059, p < 0.001).

IDO1 expression is higher in the placental microcirculation than the big chorionic plate arteries used for the above myography experiments. To examine the vascular relaxant effect of L-Trp in the placental microcirculation, we measured fetal vessel back pressure differences in response to L-Trp as a measure for the relaxation of the microvascular tone in single cotyledons from normal term placentas by *ex-vivo* placental perfusion. Perfusion of non-preconstricted cotyledons obtained from 5 different placentas with 8 mM L-Trp yielded a 13.5% median decrease (range between −16.3 and +46.5%) of the pressure measured in the artery after 40 min (a representative experiment is shown in Fig. 2a). The coefficient for the linear relationship between pressure and time was different from zero (−0.175, SE 0.020, P < 0.001), and the vessel back pressure was overall significantly decreased by 0.175 mmHg per min after attaining the stable phase (Fig. 2c). In a second set of experiments we pre-constricted the vascular bed of normal term placental cotyledons with 10 nM of U46619. After achieving stable contraction perfusion of cotyledons with 8 mM L-Trp in the presence of U46619 resulted in a 18.5% median decrease (range between −4.9 and +47.7%) of the pressure measured in the artery after 40 min (a representative experiment is shown in Fig. 2b); the coefficient for the linear relationship between pressure and time was different from zero (−0.651, SE 0.059, P < 0.001), and the vessel back pressure was overall significantly decreased by 0.651 mmHg per min after pre-constriction with U46619 (Fig. 2c).

### IDO1 expression and activity in healthy and pathologic placenta

**Figure 3:**
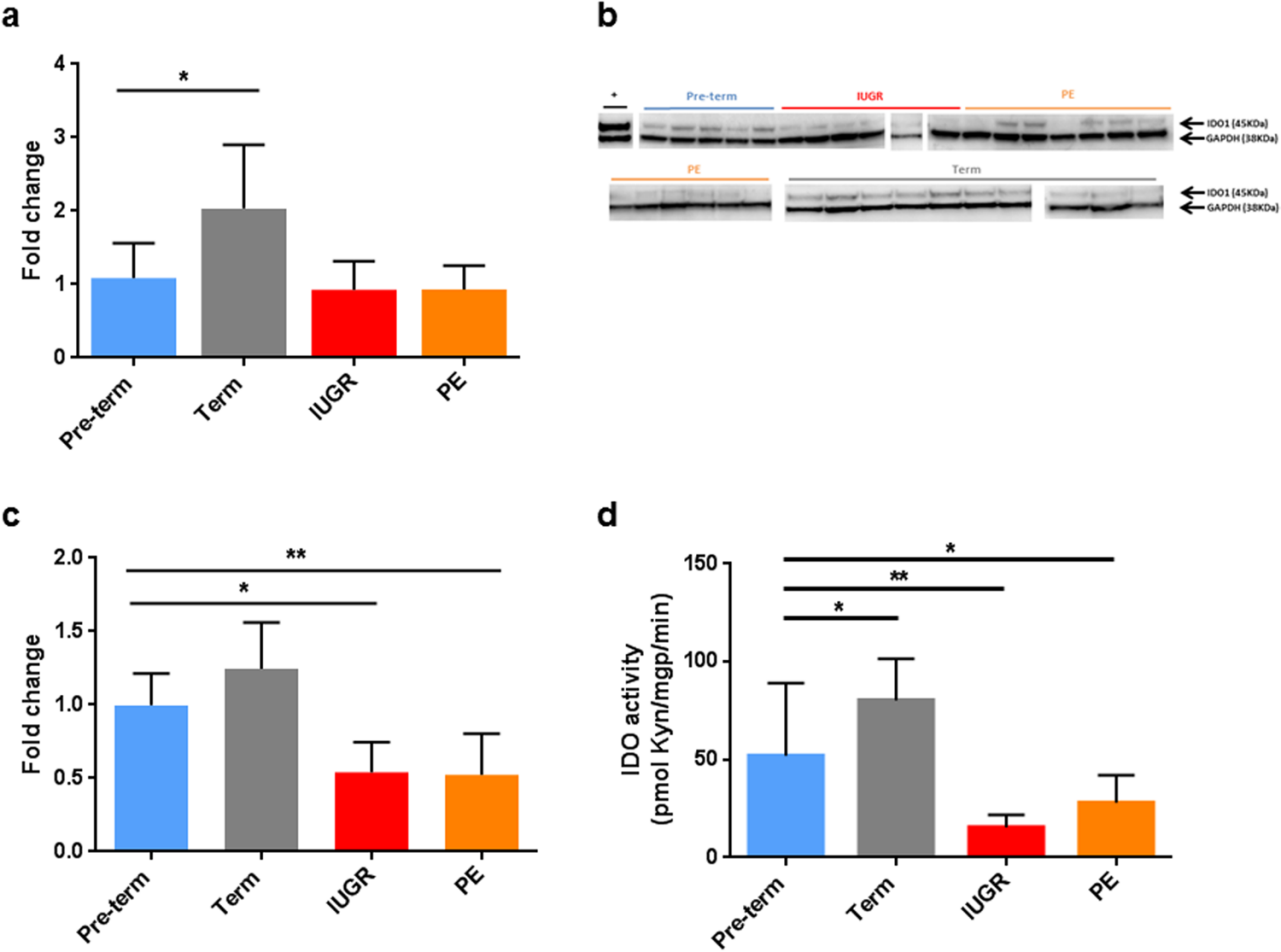
IDO1-mRNA and -protein expression, and IDO activity in chorionic tissue of normal and pathological placentas. (**a**) IDO1 mRNA in the pre-term controls (n = 5), term placentas (n = 10), IUGR (n = 6) and PE (n = 13) was analyzed by RT-qPCR. Results were normalized to the expression of the endothelial marker CD34 and calculated as a fold change relative to the pre-term control. (**b**) IDO1 Western Blot of chorionic tissue of IUGR and PE. Samples originate from pre-term controls (n = 5), term controls (n = 11), IUGR (n = 6) and PE (n = 13). The gels are grouped in pre-term control (blue line), term control (grey line), IUGR (red line) and PE (orange line). Fibroblasts stimulated with IFNγ and TNFα were used as a positive control (black line). (**c**) Densitometric analysis of IDO1 protein by Western Blot in the pre-term controls (n = 5), term placentas (n = 11), IUGR (n = 6) and PE (n = 13). The results were normalized to GAPDH protein and calculated as fold change relative to the pre-term control. (**d**) IDO activity was measured in the pre-term controls (n = 11), term placentas (n = 9), IUGR (n = 7) and PE (n = 13) by LC/MS. IDO1 activity is expressed as kynurenine formed per mg tissue protein per min. Results are given as mean ± SD. *P ≤ 0.05, **P ≤ 0.01 (Dunnett’s test in **a**, **c** and **d**).

IDO1 mRNA, protein, activity and tissue distribution were examined in banked chorionic tissues from pre-term controls (placenta praevia with a mean gestational age similar to IUGR and PE), normal term placenta, IUGR and PE (Table 1). When normalized to the endothelial marker CD34 (Fig. 3a) IDO1 mRNA was increased in term (n = 10) pregnancies compared to pre-term controls (n = 5) (P < 0.05). However, when normalized to the housekeeping gene RPL30 (Supplementary Fig. S6) we found no such difference. No differences were found in IUGR (n = 6) and PE (n = 13) tissue samples compared to pre-term controls (Fig. 3a). In contrast, IDO1 protein content was lower in the IUGR (n = 6; P ≤ 0.05) and PE (n = 13; P ≤ 0.01) samples compared to the pre-term controls (n = 5) (Fig. 3b,c). In line with protein content, IDO activity was lower in IUGR (n = 7; P < 0.01) and PE (n = 13; P < 0.05) compared to the pre-term controls (n = 11), whereas it was higher in term placenta tissues (n = 9; P < 0.05) (Fig. 3c).

**Table 1:** Clinical data pertaining to the fresh and biobanked placental samples Abbreviations: V, vaginal; C, cesarean; F, female; M, male; Y, yes; N, no. Data are mean ± SD.

	Pre-term Controls	Term Placentas	IUGR	PE
Number of samples	10	44	10	18
Maternal age (years)	33.1 ± 4.5	30.5 ± 5.6	32.4 ± 5.2	32.7 ± 7.3
Mode of delivery (V:C)	0:10	14:29*	0:10	3:15
Gestational age (weeks)	33.6 ± 1.9	39.4 ± 1.1	34.1 ± 2.4	33.7 ± 2.4
Newborns’ sex (F:M)	4:6	24:19*	5:5	13:5
Newborns’ weight (grams)	2069.2 ± 308.3	3437.7 ± 502.3	1572.8 ± 420.4	1721.8 ± 473.2
Placental weight (grams)	498 ± 64.4	651.9 ± 137.5	347.8 ± 88.4	384.7 ± 96.1
Asymmetric biometry (Y:N)			5:5	
Estimated fetal weight < 3rd percentile (Y:N)			10:0	
Arrest of growth (Y:N)			9:1	
Oligohydramnion (Y:N)			3:7	
Centralization (Y:N)			8:2	

*In one case the mode of delivery and the fetal sex are unknown.

**Figure 4:**
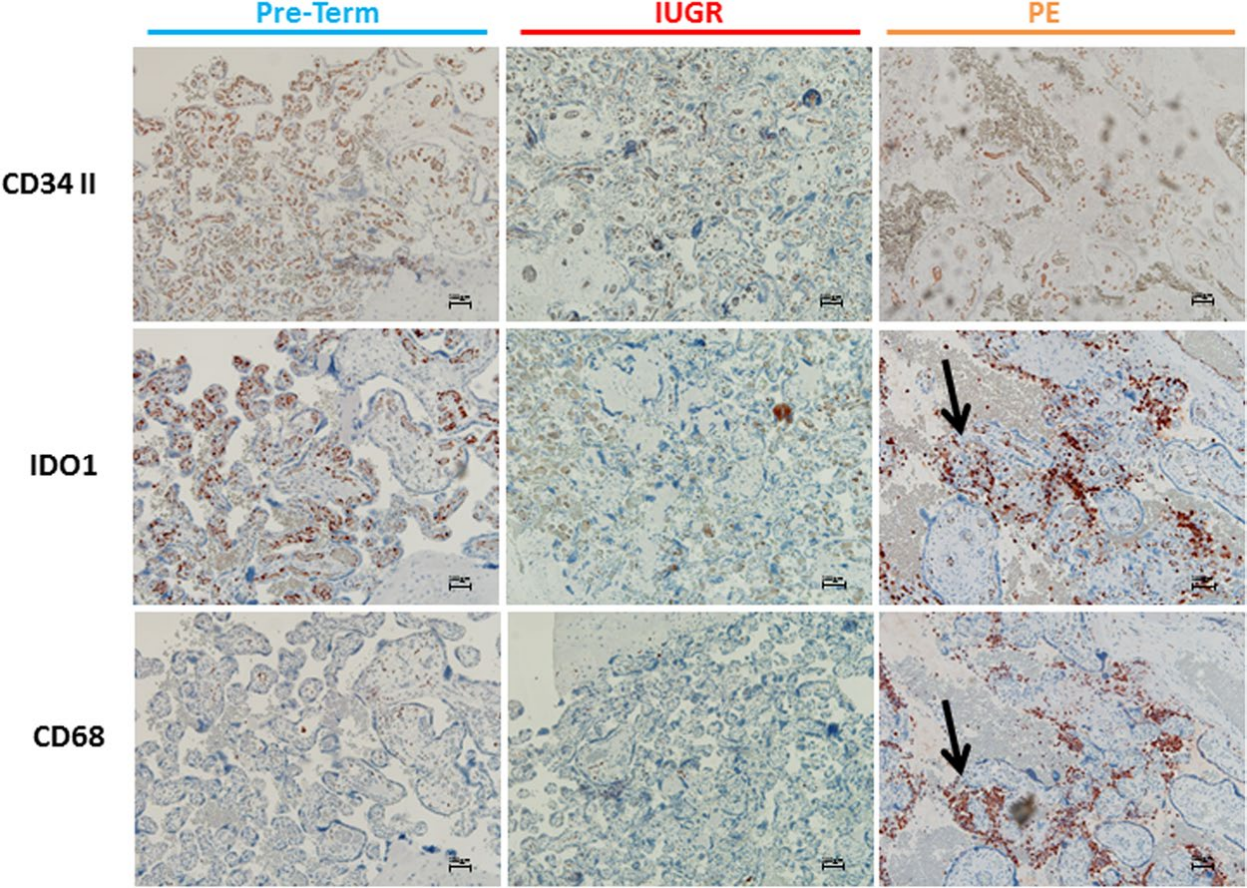
Immunohistochemical stainings of serial paraffin sections from pre-term control, IUGR and PE placental tissue. Samples were stained for CD34 II (first row), IDO1 (second row) and the macrophage marker CD68 (third row) and compared to pre-term controls (first column; blue), IUGR (second column; red) and PE (third column; orange). This PE sample contains a focus of chronic villitis and intervillositis (arrows). The scale bars represent 100 μm.

To determine whether the tissue distribution pattern of IDO1 protein was different in the chorion of IUGR and PE compared to normal term and pre-term controls, we performed immunohistochemistry on paraffin sections of banked placental tissues (Fig. 4). The expression profile of vascular endothelial IDO1 was reduced in some cases of IUGR (3 cases out of 5) and PE (4 cases out of 11) in comparison with pre-term controls (1 case out of 5). Non-endothelial localization of IDO1 was found in maternal macrophages aggregated within the intervillous spaces in areas affected by chronic villitis and intervillositis. These were found mostly in the PE group and in one pre-term control sample.

### L-Trp-elicited relaxation in rings from pathologic pregnancies

**Figure 5:**
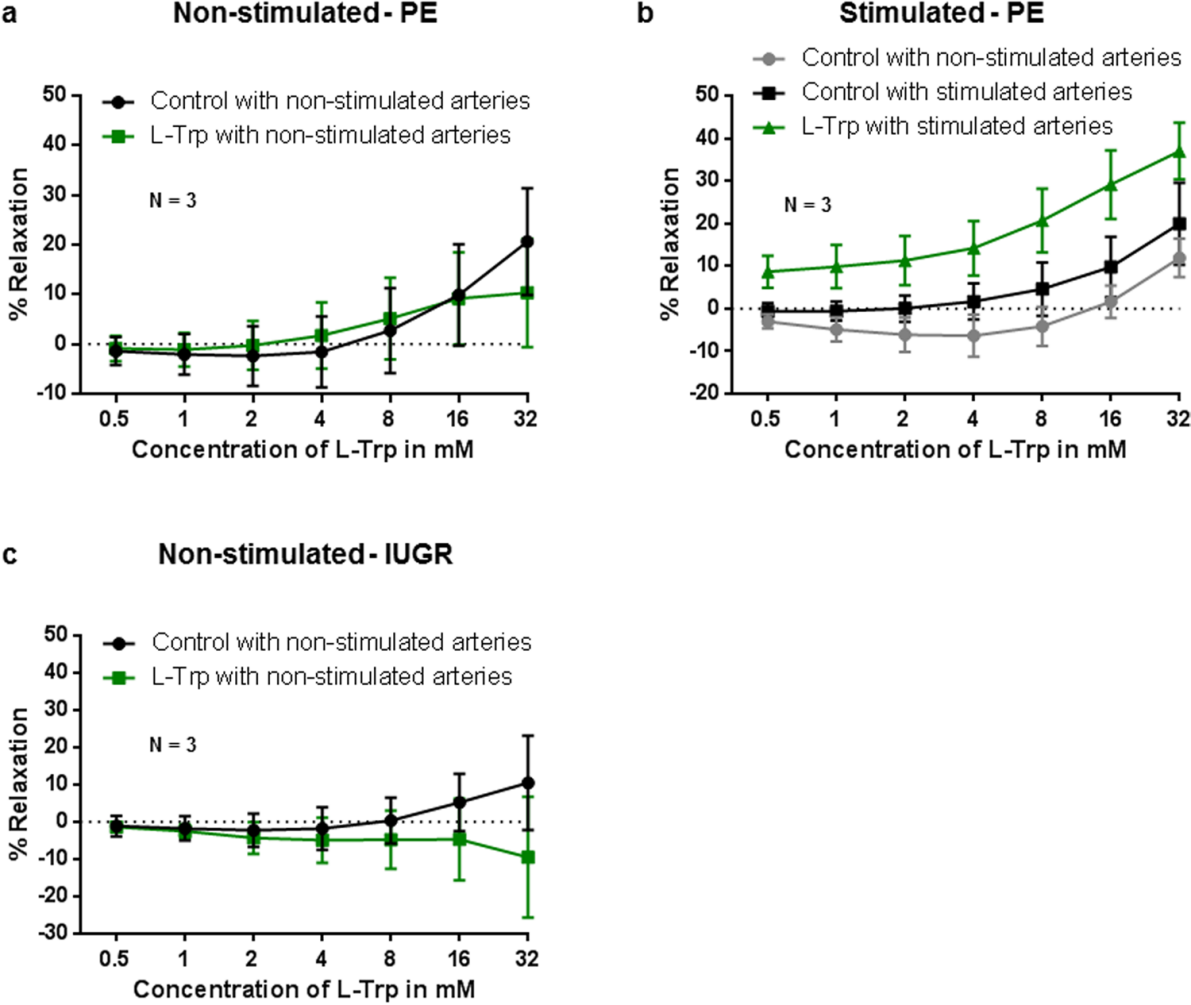
Effect of L-Trp on chorionic plate arteries from PE and IUGR pregnancies. The data show the magnitude of the L-Trp- and vehicle control- elicited relaxation of U46619-precontracted arterial rings pre-incubated overnight (**b**) or not (**a**,**c**) with TNFα and IFNγ. (**a**) P = 0.216. (**b**) ***P ≤ 0.001. (**c**) ***P ≤ 0.001. The data were determined at least in triplicates (using a minimum of 3 rings per placenta) from 3 experiments using different placentas. Results are mean ± SD (linear regression model).

L-Trp did not induce relaxation of unstimulated rings obtained from PE placentas (Fig. 5a). In contrast, in cytokine-stimulated rings L-Trp-induced relaxation compared to vehicle responses in both cytokine-stimulated and –unstimulated rings (P < 0.001). Furthermore, in stimulated PE arteries the sensitivity to L-Trp appeared slightly increased in compared to arteries from stimulated normal term pregnancies (Fig. 1b vs. Fig. 5b). Interestingly, in arterial rings from IUGR placenta L-Trp induced contraction of unstimulated rings (P < 0.001, compared to control rings) (Fig. 5c). Preliminary data from one placenta only suggest that L-Trp might induce relaxation in cytokine-stimulated arterial rings from IUGR compared to vehicle control rings (Supplementary Fig. S7).

## Discussion

The placental circulation is characterized by low vascular resistance^28,29^ and a lack of neuronal innervation^30^, suggesting that regulation of vascular tone is determined by physical and/or local factors. Our study focussed on the biological significance of the endothelial expression of IDO1 in the chorionic vasculature described earlier^13^. Apart from the placenta, constitutive endothelial expression of IDO1 has been found in some tumors^31^ (and Astrid Blaschitz, unpublished observation), whereas in other vascular beds, endothelial IDO1 is not normally expressed unless during inflammation. In this state the enzyme contributes to the regulation of the vascular tone^18^ and probably also to hypotension in human sepsis^32^. Based on studies with commercial preparations, the Trp metabolite kynurenine was originally reported to mediate relaxation of vascular tone. More recently, however, HPLC-purified kynurenine was reported to be inactive, and arterial relaxation attributed to a yet to be identified Trp metabolite^33^.

The distribution of endothelial IDO1 expression in the placenta shows a positive gradient on both the maternal and the fetal side towards the feto-maternal interface^13^. This finding implies that only a minority of the bigger arteries of the chorionic plate show IDO1 protein in their endothelium. This could explain why L-Trp did not induce arterial relaxation in this vascular bed, and why arterial relaxation was only seen after cytokine-mediated upregulation of endothelial IDO1. The fact that 1MetDTrp as well as INCB024360 blocked the effect of L-Trp implies IDO1 in the production of a vasoactive L-Trp metabolite in endothelial intact arteries^34–36^, as both of these inhibitors do not sufficiently inhibit IDO2 or TDO^34–38^.

Cytokine-stimulated intact and endothelium-denuded vessels relax similarly in response to L-Trp. Our data suggest that following cytokine stimulation, the relaxation of denuded arteries does not depend exclusively on IDO1, which is expressed in the tunica media in a few cells that remain to be characterized. There might be an increased availability of L-Trp to these cells once the endothelium is removed. It seems unlikely that this arterial relaxation is mediated by IDO2 given that the L-Trp degrading effect of this enzyme is very low compared to IDO1^37,39^, and that 50% of Caucasians lack functional IDO2 alleles^40^. A possible role of serotonin seems also unlikely as this tryptophan metabolite acts as a vasoconstrictor in placental arteries^41^.

As a means to test the possibility that L-Trp-induced arterial relaxation occurs in the microvasculature and that this in turn influences organ perfusion pressure we *ex vivo* perfused single cotyledons with L-Trp. In these experiments L-Trp caused decreased vessel back pressure in pre-constricted and non-preconstricted circulatory beds in the absence of previous cytokine stimulation. Despite the fact that the myography experiments showed that L-Trp -induced relaxation is slight in the larger chorionic plate arteries, the effect of L-Trp on decreasing vessel back pressure is more substantial and potentially suggests a greater physiological relevance, even though also in these experiments the variability of the response to L-Trp was substantial. Whilst the role of IDO1 was not directly proven in this experiment by specific blocking, we speculate that this enzyme plays a role in this response given its constitutive expression in this vasculature^13^. Also, we are reasoning that in otherwise healthy arteries with intact endothelium L-Trp does not relax. Indeed these arteries require cytokine stimulation before relaxation to L-Trp is observed, an effect which is inhibited by IDO1 inhibition. As we localized IDO1 in the endothelium of placental capillaries very early on in pregnancy^13^, it will be interesting to study to which extent smooth muscle cells and other cells, such as pericytes, are involved in L-Trp–mediated regulation of the vascular tone.

Cellular uptake of L-Trp is the pre-requisite for the effect of IDO1 a cytosolic enzyme. IDO1 has been shown to induce the expression of a L-Trp transporter in human tumor cells^42^. Recent studies indicate that L-Trp transporters can be induced by IFNγ^43^ and that 1MetDTrp does not only competitively inhibit IDO1 but also blocks L-Trp transport into the trophoblast^44^. However, our data gained by *ex vivo* placental perfusion (in the absence of IFNγ) and the results of myography experiments using INCB024360, a competitive IDO1 inhibitor that does not affect L-Trp transport^37^, renders the possibility unlikely that the vasorelaxing effect of L-Trp on cytokine-stimulated arteries is based solely on upregulation of an endothelial L-Trp transporter.

Previously we had reported increasing expression of IDO1 in the chorionic vascular endothelium during the course of pregnancy^13,14^ that correlated with increased IDO activity assessed by the kynurenine-to-Trp ratio^13^. Similar results were found in term placentas of rhesus monkeys and common marmosets^45^. Conflicting data regarding the localization of IDO1 in the placenta^46–48^ are discussed extensively elsewhere^13^. Regarding placental pathology, earlier reports^21,49^ indicate a decrease in L-Trp catabolism by placental IDO1 in IUGR and PE. In accordance with this, we found decreased IDO activity in IUGR and PE compared with pre-term controls, as determined by LC/MS. Based on semi-quantitative assessment by immunohistochemistry, a recent publication reported decreased IDO1 in villous stromal endothelial cells in placentas with PE^50^, supporting our data that show decreased IDO protein expression in PE and IUGR in comparison to gestational age-matched normal placentas. Thus, the decrease in IDO1 expression reported earlier for PE placentas^22,23^ cannot be explained solely by the fact that term placentas are an inappropriate control for PE placentas, as PE and IUGR pregnancies are associated with pre-term delivery. Indeed, we show that PE and IUGR placentas have a decreased IDO activity and express less IDO1 protein than pre-term placentas delivered. Although the IUGR and PE tissue samples showed some diversity in the extent of IDO1 protein expression, the difference to gestational age-matched controls is striking. This is particularly so when considering that in some PE placental samples part of the IDO1 protein was localized to macrophages in the foci of chronic villitis and intervillositis rather than to endothelial cells. It should be noted, however, that we cannot exclude an influence of the mode of delivery on placental expression of IDO1. In our sample this might potentially affect the difference between term and preterm placentae (see Table 1).

At the level of analysis of vascular tone, our data suggest that in PE cytokine-stimulated chorionic arteries are sensitive to low concentrations of L-Trp, possibly due to a constitutive expression of L-Trp transporters^51^. In contrast, we observed a contracting effect of L-Trp in non-stimulated arteries in IUGR. These data should be interpreted with caution, however, as gestational age–matched chorionic plate arteries were unavailable to our study.

While IDO1 mRNA did not differ between normal and pathological placentas, there were clear differences in IDO1 protein content and in functional assays. We speculate that posttranscriptional or posttranslational modifications might differentially affect IDO1 protein stability in normal and diseased placenta^52–54^. As IDO1 deficient mice have a PE phenotype^24^, our data suggest a possible pathogenic role of a deficiency in IDO1-mediated L-Trp catabolism for placenta pathology. It should be kept in mind that, whereas our study focussed on the chorionic vasculature, alterations of IDO1 expression in the endothelium of the vasculature of the decidua (where the enzyme is also constitutively expressed^13^) might as well pertain to human pregnancy pathology. Further analysis of *ex vivo* placental perfusion of IUGR and PE placentas and of the uterine and placental blood flow in patients are warranted to shed more light on this issue.

Our findings provide evidence for a novel mechanism of the regulation of the placental vascular tone, based on IDO1-mediated metabolism of L-Trp, and suggest that dysregulation of this mechanism may be involved in the pathogenesis of the pregnancy complications, such as IUGR and PE.

## Materials and Methods

### Ethical approval

The study, including the isolation of placental arterial endothelial cells, was performed in accordance with the national guidelines and approved by the Ethics Committee of the Medical University of Graz, Austria (No. 25–366 ex 12/13 and No. 21–079 ex 09/10). All participants involved in the study gave written informed consent. Samples obtained from the Biobank Graz also included informed consent.

### Patients

IUGR was defined as the presence of signs for placental insufficiency and fetal compromise (without the characteristics of PE). These signs included asymmetric fetal biometry and estimated fetal weight below the 3^rd^ centile (with confirmed gestational age by first trimester crown-rump-length measurement), oligohydramnios and centralized fetal circulation. From January 2016 on the Barcelona calculator was used for staging IUGR http://medicinafetalbarcelona.org/calc/^55^ and included cases with at least IUGR stage 1. PE was defined after week 20 of pregnancy as displaying a blood pressure higher than 140/90 mmHg (without pre-existing hypertension) and proteinuria. All patients were treated at the Department of Obstetrics and Gynaecology of the Medical University of Graz.

### Wire myography

Placentas were obtained from normal, IUGR and PE pregnancies within 30 min after caesarean section or vaginal delivery. Arterial rings (2 mm length) were cut from arteries of the chorionic plate using a calibrated eyepiece micrometre. The arterial rings were either used immediately or after overnight incubation at 37 °C in 95% air/5% CO_2_ in DMEM culture medium (Life Technologies, Paisley, UK) supplemented with 10% (v/v) FBS (Life Technologies), and stimulated with human TNFα (80 ng/mL, Peprotech, Vienna, Austria) and human IFNγ (80 ng/mL, Peprotech). For some experiments, arterial rings were denuded of their endothelium by perfusion with 0.1% (v/v) Triton X-100 (Sigma, St. Louis, MO, USA), successful denudation was confirmed by immunohistochemistry. Arterial rings were carefully mounted on two pin-supports on a myograph 620 M (Danish MyoTechnology, Aarhus, Denmark) in a modified Krebs-Henseleit buffer (Sigma Aldrich, Schnelldorf, Germany) made up in deionized water (6.9 g/L NaCl, 0.35 g/L KCl, 0.16 g/L KH_2_PO_4_, 0.141 g/L MgSO_4_, 2.1 g/L NaHCO_3_, 2 g/L D-glucose, 0.97 g/L EDTA·2H_2_O and 0.017 g/L CaCl_2_, penicillin/streptomycin 1% (w/v), pH 7.4) and continuously aerated with 5% CO_2_/95% O_2_ at 37 °C. Isometric tensions were recorded using a PowerLab and LabChart software (ADInstruments), optimal resting tension was calculated using the length-tension method as previously described^56,57^. After establishing resting tension arteries were allowed to equilibrate for 30 min. Arteries were then subject to a “wake up” protocol involving depolarising constriction to modified Krebs-Henseleit buffer containing 60 mM KCl (KPSS; 9.2 g/L KCl, 0,289 g/L MgSO_4_·7H_2_O, 0.161 g/L KH_2_PO_4_, 0.277 g/L CaCl_2_, 2.1 g/L NaHCO_3_, 0.01 g/L EDTA-Na_2_·2H_2_O, 0.991 g/L D-glucose), washout and then receptor mediated constriction using the thromboxane A_2_ analogue U46619 (10^−9^–10^−6^ M) (Sigma-Aldrich). After the “wake up” protocol contractile arteries were equilibrated in Krebs-Henseleit buffer containing indomethacin (10 μM, Sigma Aldrich), L-NAME (300 μM, Sigma Aldrich), and where necessary the IDO inhibitors 1-methyl-D-tryptophan (1 mM, 1MetDTrp; Sigma Aldrich, Schnelldorf, Germany) or INCB024360 (30 μM, MedChem Express, New Jersey, USA) for 30 min. Arteries were then constricted by a single-dose of U46619 to achieve a 50% maximal KPSS contraction. Depending on the experimental aims, arteries were either treated with a single dose of L-Trp (8 mM) or with increasing doses of L-Trp (0.5–32 mM). The extent of relaxation (% relaxation) was defined as the reversal of U46619-induced tone.

### *Ex-vivo* perfusion of a single cotyledon of the human term placenta

The *ex-vivo* perfusion system was adapted from Schneider *et al*.^58^ to study changes in the vessel back pressure of a placental cotyledon. Within 20 min after delivery of the placenta, the fetal circulation of one intact cotyledon was established by cannulating its chorionic artery and corresponding vein. Once the circuit was established, the cotyledon was flushed with pre-warmed (37 °C) perfusion medium (Earl´s buffer (6.8 g/L NaCl, 0.4 g/L KCl, 0.14 g/L NaH_2_PO_4_, 0.2 g/L MgSO_4_·7H_2_O, 0.2 g/L CaCl_2_, 2.2 g/L NaHCO_2_, all from Merck, Darmstadt, Germany), supplemented with 10 g/L dextran FP40 (Serva, Heidelberg, Germany), and 2 g/L D-glucose (Merck). The cotyledon together with surrounding tissue was cut and carefully positioned into the pre-warmed perfusion chamber. Perfusion medium kept at 37 °C was connected to the fetal artery cannula where a micro-catheter pressure sensor (Millar, Houston, Texas, US) was also inserted to monitor in real time the pressure changes. The fetal circulation was gassed with 95% N_2_, 5% CO_2_ through a gas exchanger (Living Systems, St. Albans, VT, US). A continuous fetal artery influx of 4 mL/min was applied by a magnetic pump (Codan, Salzburg, Austria). In order to establish the maternal circulation, three rounded needles were inserted and fixed into the intervillous space of the perfused cotyledon. A constant volumetric flow rate of 8 mL/min was administered to the maternal circulation; the medium was gassed with 5% CO_2_, 20% O_2_ and 75% N_2_. After a pre-stabilization period of at least 10 min, (i) either 8 mM L-Trp (Sigma Aldrich, Steinheim, Germany) (ii) or 10 nM U46619^59^, attaining a plateau of pre-contraction for at least another 10 min, followed by 8 mM L-Trp (in the continued presence of 10 nM U46619) were applied to the fetal reservoir so ensuing pressure changes could be studied.

Only experiments with a fetal flow recovery of at least 85% within the first 30 min were continued for analysis according to defined quality criteria^60^. Perfusates from maternal and fetal circulation were collected every 30 min during the experiment in order to measure the metabolic and oxygenation status (pO_2_, pCO_2_, pH, lactate production and glucose consumption) of the perfused cotyledon by a blood gas analyser (Radiometer, Copenhagen, Denmark).

### Isolation and culture of placental arterial endothelial cells (PLAECs)

Human term placentas from uncomplicated pregnancies were obtained under informed consent after vaginal delivery. After removal of the amnion, arterial chorionic blood vessels were resected from the fetal surface of the chorionic plate as previously published^61^. After removal, arteries were washed with Hank’s balanced salt solution (HBSS) (Life Technologies, Paisley, UK) to remove residual blood. PLAECs were then isolated by perfusion of vessels with DMEM containing 185 U/mL collagenase type II (Sigma, St. Louis, MO, USA), 2 mg/mL bovine serum albumin (Sigma) 0.5 mM CaCl_2_ (Roth, Karlsruhe, Germany), 1 unit amino acids 50x (PAA, Pasching, Austria), 1 unit amino acids 100x (PAA), 0.01 unit vitamin 1x (PAA) and 5% FBS, pre-warmed to 37 °C. The perfusion time was limited to 8 min to avoid contamination with non-endothelial cells. Detached cells were collected in FBS. Finally, the cell suspension obtained was centrifuged (200 × *g* for 5 min), and the pellet re-suspended with EGM-MV medium (Lonza, Walkersville, MD, USA). The cells were plated on culture plates pre-coated with 1% (v/v) gelatin (Sigma-Aldrich, Schnelldorf, Germany). The purity of the cultures was assessed by staining for the specific endothelial marker von Willebrand factor (1:8000, A0082, Dako, Glostrup, Denmark) and the absence of fibroblast specific antigen (1:2000, DIA−100, Dianova, Hamburg, Germany), smooth muscle actin (1:600, M0851, Dako) and desmin (1:1000, Dako). Only PLAECs cultures with a purity of > 99% were used for the present study. Cells were used only between the third and the sixth passage.

### Immunohistochemistry

Tissues were fixed between 24 h and 3 days in 4% (w/v) buffered formalin (Sanova, Vienna, Austria) at room temperature and embedded in paraffin. 5 μm sections were cut on a rotation microtome, mounted on Superfrost^+^ glass slides (Sanova) and dried over-night on a 50 °C hot plate. Slides were then deparaffinised with Tissue Clear (Sanova) and rehydrated followed by heat induced antigen retrieval buffer at pH 9 (Leica Biosystems, Vienna, Austria) under pressure at 120 °C for 7 min, cooled down for 20 min and washed in distilled water. Endogenous peroxidase activity was blocked by 10 min exposure to peroxidase-blocking solution (Dako, Carpinteria, CA), washed and then application of Ultra-V block (Thermo Scientific, Cheshire, UK) for 5 min. Samples were incubated first with anti-IDO1 antibody (IgG1 1 μg/mL, generously provided by O. Takikawa^9^) for 45 min at room temperature and then 10 min with primary antibody enhancer (Thermo Scientific). Bound antibodies were detected using a polyvalent horseradish-peroxidase polymer system (Thermo Scientific) for 15 min followed by 10 min incubation with 3-amino-9-ethylcarbazole (Thermo Scientific). All samples were counterstained with Mayer’s hematoxylin and aqueously mounted with Kaiser’s glycerol gelatine (Merk, Damstadt, Germany). Washing steps were performed with TBS/0.05% (v/v) Tween 20 (Merck). Irrelevant mouse anti-Aspergillus niger IgG1 (1 μg/mL, Dako) was used as a negative control antibody. Anti-CD34 class II (0.04 μg/mL, Dako) was used for staining vascular endothelium, anti-CD68 (0.025 μg/mL; Thermo Scientific) for staining macrophages.

### Fixation of cell pellets

Accutase-detached cells were re-suspended in EGM-MV medium (Lonza, Walkersville, MD, USA) and centrifuged (630 × *g* for 5 min), the supernate was carefully removed. The pellet was transferred into a 1.5 mL Eppendorf tubes, re-suspended and fixed in 1 mL 4% paraformaldehyde (PFA) for 30 min at room temperature. After two washes with PBS (630 × *g* for 5 min), the pellet was re-suspended in 5% (v/v) gelatine (Merck, Darmstadt, Germany) for 30 min at 37 °C. Following centrifugation at 37 °C, the pellet was placed overnight at 4 °C and then fixed for an hour at 4 °C by adding 4% PFA. Once the PFA was removed, the pellet was carefully placed in a new vessel with 4% PFA and incubated overnight at 4 °C. The pellet was then washed with PBS and embedded in paraffin as mentioned above.

### Protein isolation and quantification

Total cell or tissue protein lysates were prepared in RIPA buffer (Sigma Aldrich, St. Louis, MO, USA) containing protease inhibitors (Roche, Mannheim, Germany). Protein concentration was determined according to the Lowry method.

### Western blotting analysis

Cell and/or tissue lysates were mixed with LDS sample buffer (4×) (Life Technologies, Carlsbad, CA) and sample reducing agent (10×) (Life Technologies). Samples were then denatured at 70 °C for 10 min. Equal amounts of total protein (25 µg/well) were loaded onto 10% Bis-Tris SDS-PAGE gels (Life Technologies) and resolved at 120 V for approximately 75 min. Proteins were transferred to a nitrocellulose membrane (Life Technologies) and nonspecific binding sites blocked for 30 min with blocking solution (Life Technologies). After blocking, the membranes were incubated by the anti-IDO1 antibody^9^ (1 μg/mL) mixed together with an anti-GAPDH antibody (Novus Biologicals; 50 ng/mL) on a shaker overnight at 4 °C. Membranes were washed with wash solution (Life Technologies) and incubated with the appropriate secondary antibody solution conjugated with horseradish peroxidase (Life Technologies). After washing, membranes were visualized using an enhanced chemiluminescent imaging system (Life Technologies).

### Semi-quantitative polymerase chain reaction

Total RNA was isolated according to manufacturer’s instructions using peqGOLDTriFast (VWR, Erlangen, Germany). cDNA was synthetized from 2 μg of RNA in the presence of random primers and MultiScribeTM reverse transcriptase (Applied Biosystems, Cheshire, UK). IDO1 specific fragments (297 bp) were amplified (62 °C, 35 cycles) by PCR and detected by 2% (w/v) agarose gel electrophoresis.

### Real time polymerase chain reaction (RT-qPCR)

Total RNA from placental tissue was isolated using the peqGOLD TriFast reagent (VWR) according to the manufacturer’s protocol. For RNA extraction, small tissue pieces were cut and homogenized using a tissue homogenizer and the TriFast reagent. RNA quality was assessed on 1.5% (w/v) denaturing agarose gels (Biozym, Vienna, Austria) and stained with GelRed™ Nucleic Acid Gel Stain (Biotium, Fremont, CA). RNA quantity and purity were assessed using a NanoDrop1000 Spectrophotometer (Thermo Scientific). After the quality check, 2 µg of total RNA was reverse transcribed with the High-Capacity cDNA Reverse Transcription Kit (Applied Biosystems, Foster City, CA). Reverse transcription was performed with and without reverse transcriptase to check for a possible contamination with genomic DNA. cDNA was employed for quantitative real-time PCR using TaqMan Gene Expression Assays (Applied Biosystems) for human IDO1 [Hs00158027_m1], human CD34 [Hs00990732_m1] and ribosomal protein L30 (RPL30)[Hs00265497_m1] and the TaqMan Universal PCR Mastermix (Applied Biosystems). cDNA and kit compounds were mixed in a total volume of 20 μL per well (96 well plates, Thermo Scientific) according to the manufacturer’s instructions and amplified using a Bio-Rad CFX96 Real-Time PCR System (Bio-Rad, Vienna, Austria). Ct values were automatically generated by the CFX Manager 2.0 Software (Bio-Rad). Relative quantification of gene expression was calculated by standard ∆∆Ct method using the expression of CD34 or RPL30. Data are presented as mean of 2−∆∆Ct values.

### IDO activity analyzed by liquid chromatography – tandem mass spectrometry (LC-MS/MS)

IDO activity in placental tissue homogenates was measured as the amount of kynurenine formed from L-Trp using the ascorbate/methylene blue assay as previously described^62^ with modifications. Placental tissue homogenates were generated by placing frozen placental tissue (cut into small pieces on dry ice) in 500 μL of 100 mM phosphate buffer pH 7.4 containing 2x protease inhibitor cocktail (Roche Diagnostics, Sydney, Australia) and homogenizing the tissue using a Heidolph homogenizer. Homogenates were centrifuged at 17,000 x *g* for 15 min at 4 °C and the supernate used for IDO activity assay. The standard assay mixture (in 300 μL final volume) consisted of 100 mM phosphate buffer, pH 7.4, containing 200 μL supernate, 200 μM L-Trp, 10 mM ascorbic acid, and 0.2 mg/mL catalase. The reaction was initiated by adding 15 μL 500 μM methylene blue (25 μM final concentration) to the above reaction mixture, followed by incubation for 30 min at 37 °C. Aliquots (100 μL) were removed at 0 min (before incubation at 37 °C) and at 30 min and the reaction stopped by the addition of cold trichloroacetic acid (4% (w/v) final concentration). Mixtures were stored on ice for 15 min and then incubated at 65 °C for a further 15 min to convert N-formyl-kynurenine to kynurenine before centrifugation at 17,000 x *g* for 15 min at 4 °C. To 25 μL supernate, equal volume of 1 M phosphate buffer, pH 7.4 was added, the mixture vigorously mixed for 30 s and then centrifuged at 17,000 × *g* for 5 min at 4 °C. Five microliters of the supernate were then injected onto an Agilent 1290 UHPLC system connected to an Agilent 6490 triple-quadrupole mass spectrometer equipped with an electrospray ionization source (ESI) operating in positive ion mode. Analytes were separated on a 5 μm Luna C18 (2) column (30 × 2.1 mm; Phenomenex, USA) by gradient elution using 0.1% acetic (mobile phase A) and 0.1% acetic acid in acetonitrile (mobile phase B) at 0.15 mL/min. The gradient consisted of 0–5% mobile phase B from 0 to 6 min, 5–100% from 6 to 8 min. Mass spectrometer parameters were as follows: gas temperature = 250 °C; gas flow = 20 L/min; nebulizer pressure = 241.3 kPa); sheath gas heater = 325 °C; sheath gas flow = 12 L/min; capillary voltage = 3,500 V.

Detection of kynurenine was by multiple reaction monitoring (MRM) in positive ion mode using the above general mass spectrometry parameters with fragmentor voltage at 380 V and cell accelerator voltage at 5 V. The largest fragment ion generated by collision-induced dissociation of the [M + H]^+^ ion were used for quantification with the following transition (parent ion → fragment ion) m/z 209 → 146 with CE = 10 V. Kynurenine was quantified against a standard curve of authentic commercial standard obtained from SigmaAldrich (USA).

### Statistics

Statistical analyses were performed using GraphPadPrism (version 6.01) and R (version 3.3.2). Data were expressed as mean ± standard deviation. Normality of distribution was assessed by Kolmogorov-Smirnov and Shapiro-Wilk tests. Unpaired data were analysed with Dunnett’s test for comparisons against one reference treatment, paired non-parametric data with Wilcoxon signed-rank test and paired parametric data with *t*-test. The relaxation percentages were related to the concentration (mM) using a linear regression model on original scale including treatments, a linear and a quadratic component for the concentration, an interaction term for treatments and concentration as fixed effects and additionally intercept and concentration as random effects. We will state that two treatments have a different effect if the coefficients of the linear component differ. P-values less than 0.05 were considered statistical significant.

### Data Availability

The datasets generated during and/or analysed during the current study are available from the corresponding author on reasonable request.

## Electronic supplementary material

Supplementary Figures online
