# Supplementary material for: Endothelial indoleamine 2,3-dioxygenase-1 regulates the placental vascular tone and is deficient in intrauterine growth restriction and pre-eclampsia
Source: Sci Rep. 2018 Apr 3;8:5488. doi: 10.1038/s41598-018-23896-0 (PMC5883010; doi:10.1038/s41598-018-23896-0)
Supplement: Supplementary file 1 — Supplementary Figures online [file 41598_2018_23896_MOESM1_ESM.pdf]

Endothelial indoleamine 2,3-dioxygenase-1 regulates the placental vascular tone and is deficient in intrauterine growth restriction and pre-eclampsia

Pablo Zardoya-Laguardia[1], Astrid Blaschitz[1], Birgit Hirschmugl[2], Ingrid Lang[1], Sereina A. Herzog[3], Liudmila Nikitina[1], Martin Gauster[1], Martin Häusler[2], Mila Cervar-Zivkovic[2], Eva Karpf[4], Ghassan J. Maghzal[5,6], Chris P. Stanley[5], Roland Stocker[5,6], Christian Wadsack[2], Sasa Frank[7], and Peter Sedlmayr[1*]

Supplementary Figures

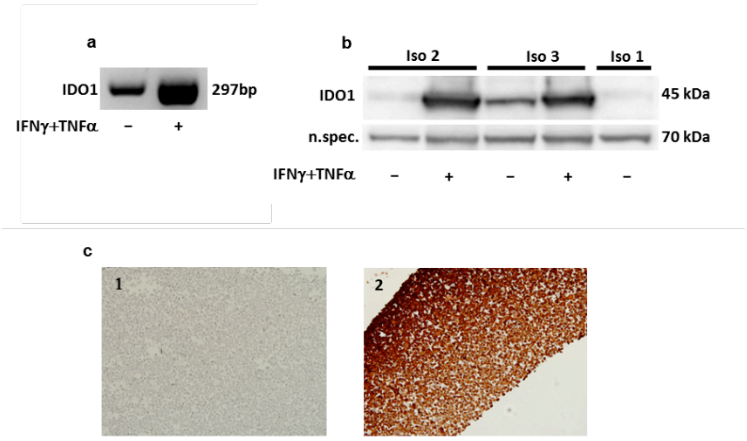

Supplementary Figure S1. IDO1 expression in placental (chorionic plate) arterial endothelial cells (PLAECs). (a) IDO1 mRNA expression in PLAECs by qPCR in the absence or presence of IFNγ and TNFα for 48 h. A 297 bp PCR-fragment of IDO1 was evaluated by gel-electrophoresis on 2% agarose gel in a single experiment. **(b)** IDO1 protein expression in PLAECs isolated from non-pathological term placentas, determined by Western blotting. PLAECs from 3 isolations (Iso 1-3) were incubated in the absence or the presence of IFNγ and TNFα. **(c)** Immunohistochemical staining of cell pellets of PLAECs without **(c1)** or with **(c2)** 24 h - stimulation with IFNγ and TNFα. Data regarding the cell pellets are representative of two independent experiments.

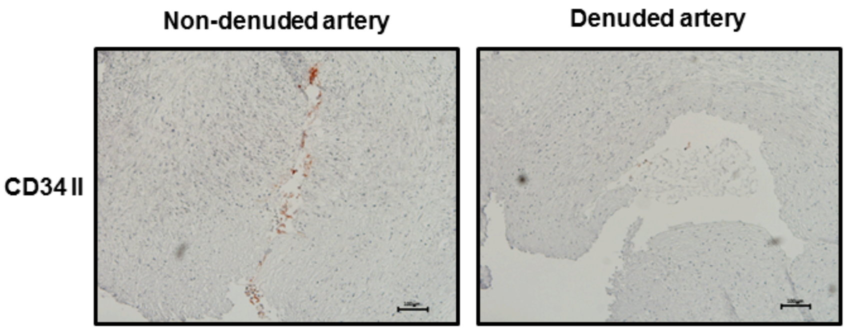

Supplementary Figure S2. Immunohistochemical control for the efficiency of endothelial denudation. Given is an example of staining for the endothelial marker CD34 before and after denudation. The scale bar represents 100 μm.

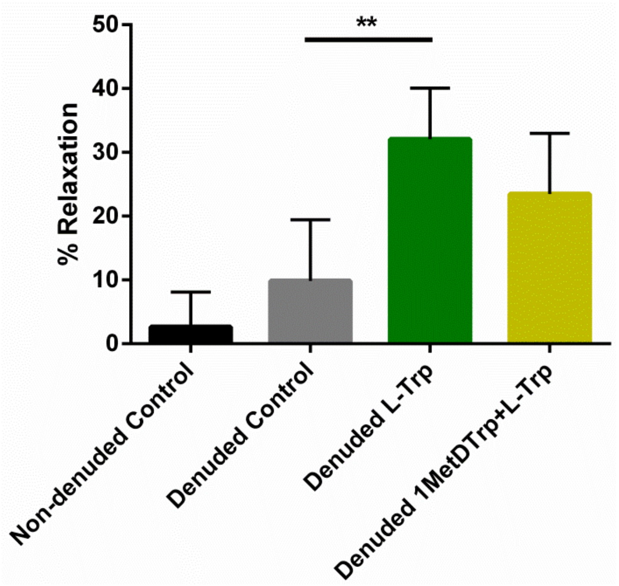

Supplementary Figure S3. Analysis of the contribution of IDO1 to the relaxing effect of L-Trp on denuded chorionic arteries stimulated with IFNγ and TNFα. Shown is the relaxation following the application of 8 mM L-Trp to non-denuded or denuded stimulated normal chorionic plate arteries in the presence (1MetDTrp+L-Trp) or absence (L-Trp) of 1mM 1MetDTrp, in comparison with control (Control). The data were measured at least in duplicates and are the results from 3 independent experiments using different placentas. They are indicated as mean ± SD; **P ≤ 0.01. The impact of blocking of IDO1 with 1-MetDTrp is not significant.

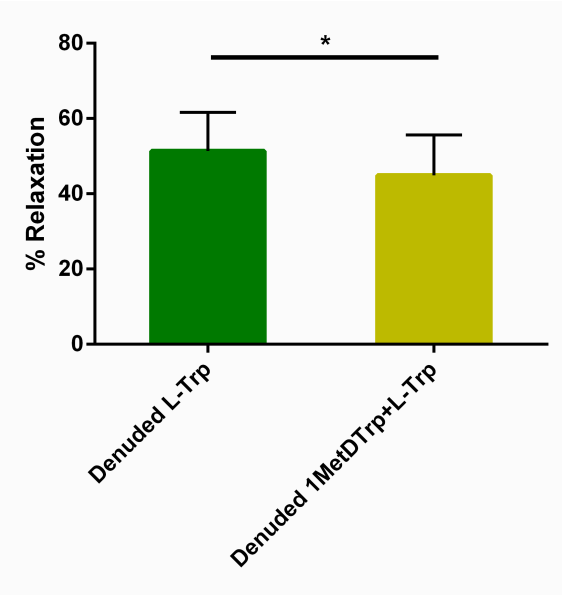

Supplementary Figure S4. Results of further experiments (planned at a statistical power of 90%) regarding the contribution of IDO1 to the relaxing effect of L-Trp on denuded stimulated chorionic arteries. Shown is the relaxation following the application of 8 mM L-Trp to denuded stimulated normal chorionic plate arteries in the presence (1MetDTrp+L-Trp) or absence (L-Trp) of 1 mM 1MetDTrp. The data were measured in 24 arterial rings per group and are the results from 4 experiments using different placentas. They are indicated as mean ± SD. *P ≤ 0.05.

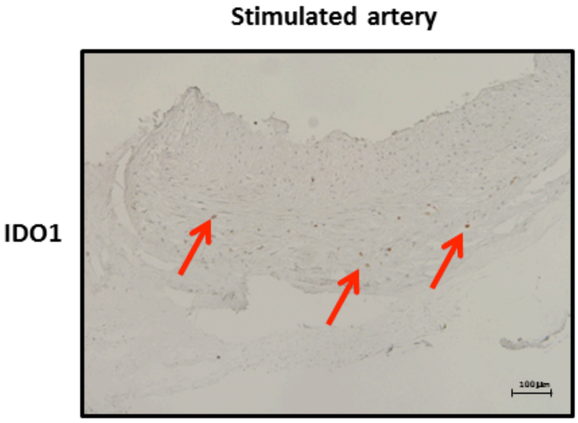

Supplementary Figure S5. Immunohistochemical staining of IDO1 in stimulated arteries from normal pregnancies. Arrows indicate IDO1-positive cells in cytokine-stimulated

arteries from the chorionic plate of a normal placenta. The smooth muscle layer is on the whole IDO1-negative. The scale bar represents 100 μm.

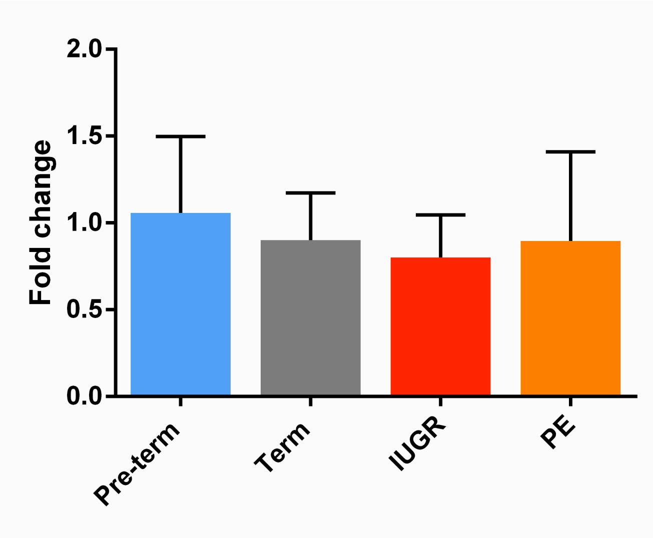

Supplementary Figure S6. IDO1-mRNA expression in chorionic tissue of normal and pathological placentas. IDO1 mRNA levels in the pre-term controls (n = 5), term placentas (n = 10), IUGR (n = 6) and PE (n = 13) were analysed by RT-qPCR. Results were normalized to the expression of the housekeeping gene RPL30 and calculated as a fold change relative to the pre-term control. Results are given as mean ± SD.

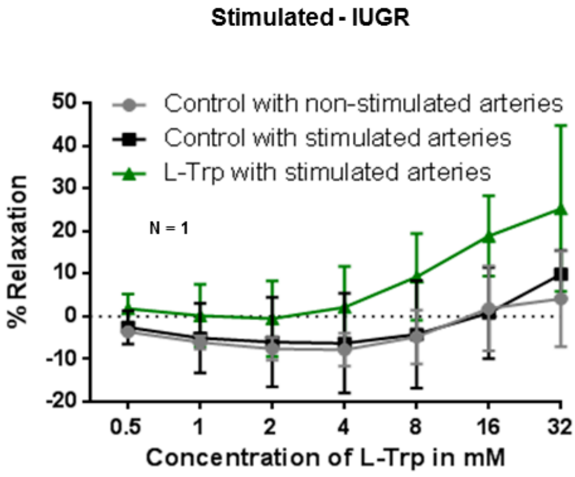

Supplementary Figure S7. Effect of L-Trp on chorionic plate arteries from a IUGR pregnancy. Preliminary data from 1 experiment showing the magnitude of the L-Trp- and

vehicle control- elicited relaxation of U46619-precontracted arterial rings pre-incubated overnight with TNFα and IFNγ. The data were determined in quadruplicates (using 4 arterial rings). Results are mean ± SD.
